# Vertical migration by bulk phytoplankton sustains biodiversity and nutrient input to the surface ocean

**DOI:** 10.1038/s41598-020-57890-2

**Published:** 2020-01-24

**Authors:** Kai Wirtz, S. Lan Smith

**Affiliations:** 1 Institute of Coastal Research, Helmholtz Centre Geesthacht, Geesthacht, Germany; 2 Earth SURFACE System Research Center, Research Institute for Global Change, JAMSTEC, Yokosuka, Japan

**Keywords:** Behavioural ecology, Biodiversity, Biogeochemistry, Biooceanography, Community ecology, Ecological modelling, Biogeochemistry, Marine biology

## Abstract

Phytoplankton subsumes the great variety of unicellular photoautotrophs that perform roughly half of Earth’s primary production. They achieve this despite their challenging oceanic habitat, with opposing vertical gradients of nutrients (which often limit their growth near the surface) and light (which becomes limiting with increasing depth). Most phytoplankton species are commonly assumed to be incapable of moving actively between the zones of light and nutrient availability, which are separated vertically by from 30–120 m. Here we propose that a considerable fraction of phytoplankton vertically traverse these gradients over time scales from hours to weeks, employing variations of a common migration strategy to acquire multiple resources. We present a mechanistic Lagrangian model resolving phytoplankton growth linked to optimal migration behaviour and demonstrate unprecedented agreement of its calculated vertical CHL-a distributions with 773 profiles observed at five prominent marine time-series stations. Our simulations reveal that vertically cycling phytoplankton can pump up enough nutrient to sustain as much as half of oceanic Net Primary Production (NPP). Active locomotion is therefore a plausible mechanism enabling relatively high NPP in the oligotrophic surface ocean. Our simulations also predict similar fitness for a variety of very different migration strategies, which helps to explain the puzzling diversity of phytoplankton observed in the ocean.

## Introduction

### Phytoplankton as active drifters

Primary production by phytoplankton mediates about half the global carbon and nitrogen cycles, despite the rarity of favourable conditions for their growth in the surface ocean. Nutrients are typically scarce and light is only plentiful above the chemocline depth, which varies from 60–180 m. Meanwhile, near the nutrient rich chemocline, low light intensity severely limits photosynthesis. The two zones where either nutrients or light become replete are separated by about 30–120 m, a distance that challenges the locomotion ability of most phytoplankton species, which are classically assumed to be passive drifters. This picture of drifters has been corrected through evidence of active and fast migration through the water column at 20–100 md${}^{-1}$ for a few phytoplankton groups, such as diatom mats^1,2^ and cyanobacteria^3^ in the open ocean, and for dinoflagellates in coastal waters^4^. Fast migrators can synthesize carbohydrates in the sunlit upper layers^5^ and access nutrients at depth, a strategy which is traceable through diurnal variations in their vertical distribution maxima and nutrient ratios^6^. This constitutes an effective adaptation to the vertical separation of light and nutrients^7^. Modelling studies have verified its potential to enhance the growth of various phytoplankton taxa, including mats of the diatom *Rhizosolenia*^8^, dinoflagellates^9^, and nitrogen-fixing *Trichodesmium* colonies^10,11^.

However, the majority of phytoplankton species move relatively slowly, if at all. Both fast movers and slow travelers use either buoyancy regulation or active swimming. A total of 59% of non-silicified species are flagellated^12^ and thus capable of active locomotion. Members of the widespread genus *Synechococcus*, which are generally believed non-motile because lacking flagella, can actively swim at speeds of 1.5–2 m/d^13,14^. Not only some cyanobacteria but most diatoms can regulate excess cell density, thereby creating positive or negative buoyancy. Slow movement has in the past been linked to the exploitation of micro-scale nutrient hotspots, but that hypothesis has recently been questioned as a common strategy requiring motility^15^. Therefore, the adaptive significance of small to moderate sinking/ascending or swimming velocities of about 0.5–10 m/d (e.g., as typically reported for flagellates^16^ or intermediate size diatoms^17,18^) is still unclear. These velocities are too slow to allow migration between the surface and the chemocline on a daily timescale. However, observation-based estimates of migration periods were a few days, even for the large mat-forming diatom *Rhizosolenia*^1^, and species-specific modeling studies have noted that migration may occur on timescales longer than daily, in response to various environmental or physiological cues^9,11^.

We suggest that slow vertical migration on time spans of a week or so could allow cells to enhance their growth by accessing light near the surface and nutrients near the nutricline. Furthermore, we add that a non-negligible part of the phytoplankton community employs such slow, long-distance migration. These slow travelers would have to maintain growth rates below 0.2 or 0.1 d${}^{-1}$, which seems plausible because they experience strong limitation by either light or nutrients, or by the combination of both. Observed community growth rates at the lower part of the euphotic zone are generally below 0.1d${}^{-1}$, as reported for the eastern equatorial Pacific^19^ or off California^20^. Independent of fast migration over short periods or slow travelling over longer periods, this strategy requires flexibility in internal C:N:P stoichiometry. This has been observed across all size classes^21,22^, even for the cyanobacteria *Synechococcus*^23,24^.

Our study investigates two immediate consequences of widespread cyclic vertical migration for both phytoplankton ecology and biogeochemical cycling. The first connects the diverse mobility capabilities—from no, slow to fast migration—to the richness in phytoplankton species composition found throughout the euphotic zone of the ocean. Second, active locomotion in combination with intracellular storage of, for example, carbohydrates and nutrients spatially decouples biosynthesis from the availability of nutrients and light. Ascending cells with high nutrient-to-carbon ratios and descending cells with low ratios generate a net upward flux of organic nutrients, which the microbial loop recycles into an effective flux of bio-available inorganic nutrients. If operated by bulk phytoplankton rather than just a few species, this active biological nutrient pump would constitute a powerful mechanism to sustain the marine cycles of carbon, nitrogen, and other elements, despite the unfavorable conditions typical of the surface ocean.

**Figure 1:**
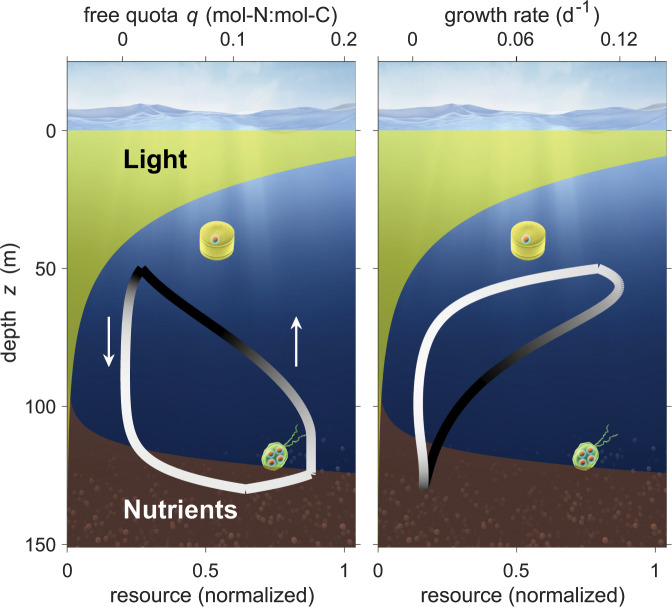
Vertical distributions of light (green shading) and nutrients (brown shading indicating the chemocline) in the upper ocean, which frame physiological changes in phytoplankton. (**a**) Intracellular free quota $q$ during a vertical oscillatory migration in the depth range ${z}_{C}\pm \delta z$ with center position ${z}_{C}$ = 90 m and half amplitude $\delta z$ = 40 m. Arrows indicate the swimming/floating direction. History dependence in the depth profile of $q$ creates a hysteresis curve. Shading of this quota hysteresis curve reflects the instantaneous growth rate $P$ (white for low, black for high). (**b**) Hysteresis curve of photosynthesis (growth) rate with shading indicating the quota level (white for low, black for high).

To test our hypothesis and study its consequences, we develop a novel modeling approach that resolves the mobility- and history-dependent physiology of unicellular autotrophs. Our model partitions the phytoplankton community into an immobile and an actively moving fraction. The mobile community consists of cells migrating down—and upwards, thereby changing their internal nutrient-to-carbon stoichiometry. The nutrient-to-carbon quotas of cells passing the chemocline increase while quotas decline in the upper sunlit layers due to photosynthetic carbon assimilation (Fig. 1). By maintaining low growth rates below 0.1 d${}^{-1}$ during most of the passage, a cell or its daughter cells can migrate up to 100 m while depleting internal nutrient stores. Our approach thus implies that cells at any given depth have either high or low nutrient quotas and correspondingly different growth rates, as they move either from or towards the chemocline. This history dependence creates a hysteresis curve in physiological variables, for which we derive an analytical model solution. Our model calculates the physiological dynamics and growth rate of mobile cells averaged over the entire cycle as a function of a variable migration strategy, characterised in terms of migration center (mean depth), amplitude, and speed. An optimization algorithm then identifies the set of strategies that approximately maximizes average growth rate during the migration cycle.

Under the assumptions of steady-state and density dependent mortality, (near-)optimal growth rates translate to an estimate for the total biomass concentration of mobile phytoplankton. This biomass, the migration traits, and the physical mixing rate determine vertical biomass distributions of the mobile fraction, to which we add the profile of drifters as estimated from surface CHL and chemocline depth. The resulting total vertical CHL distributions are compared to long-term data from five marine time-series stations, which are selected to cover a wide range of light, temperature, and nutrient regimes: the Bermuda Atlantic Time-Series (BATS), Hawaii Ocean Time-series (HOT), subarctic west Pacific (K2), subtropical west Pacific (S1), and the Gotland Deep (GD) in the Baltic Sea (Table S1).

## Results

**Figure 2:**
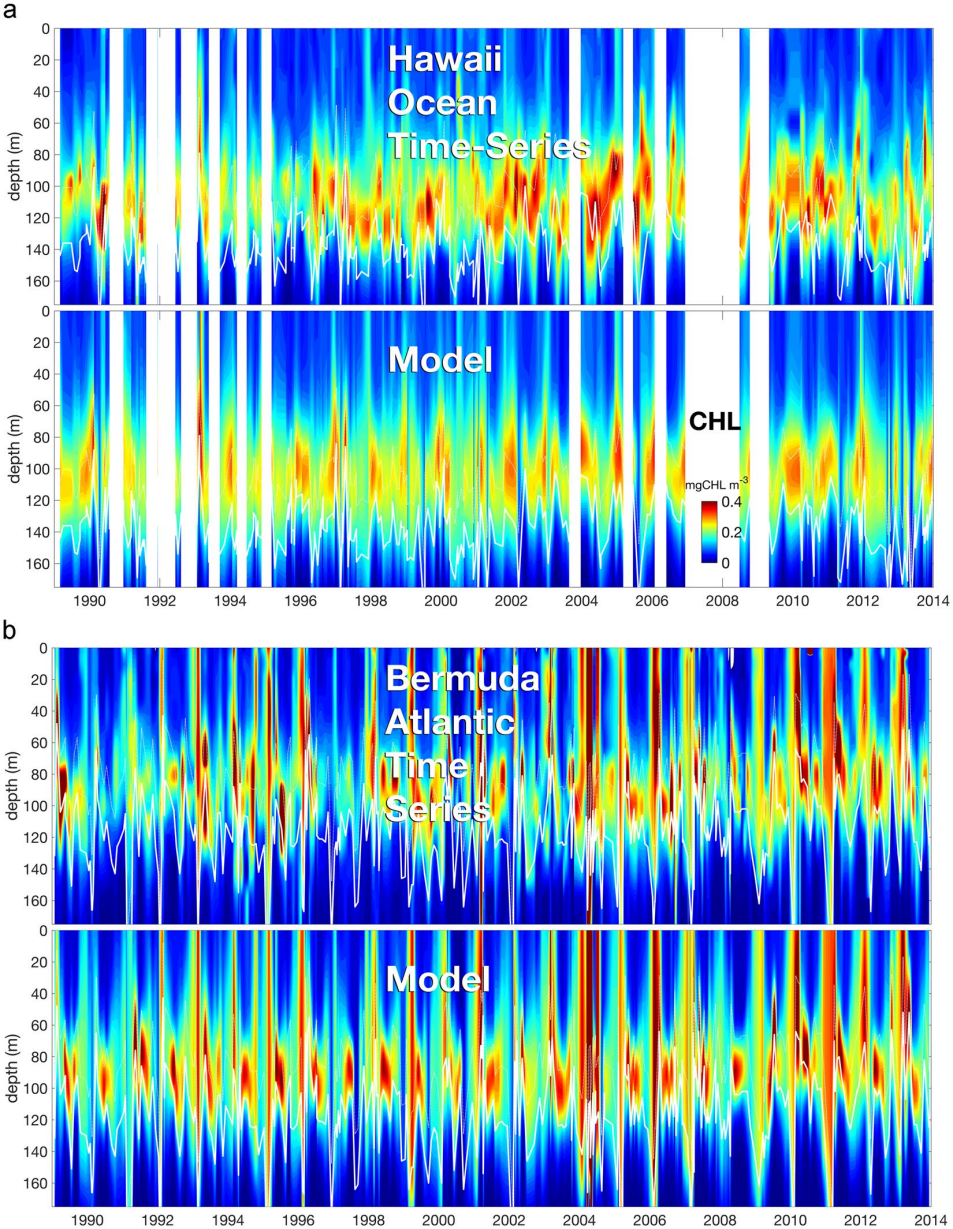
Contour plots of depth resolved chlorophyll from 1989–2014 observed at the Bermuda Atlantic Time Series (BATS, http://bats.bios.edu) and the Hawaii Ocean Time-Series (HOT, http://hahana.soest.hawaii.edu/hot/hot-dogs), both compared to model simulations. White lines show variations in reconstructed chemocline depth.

**Figure 3:**
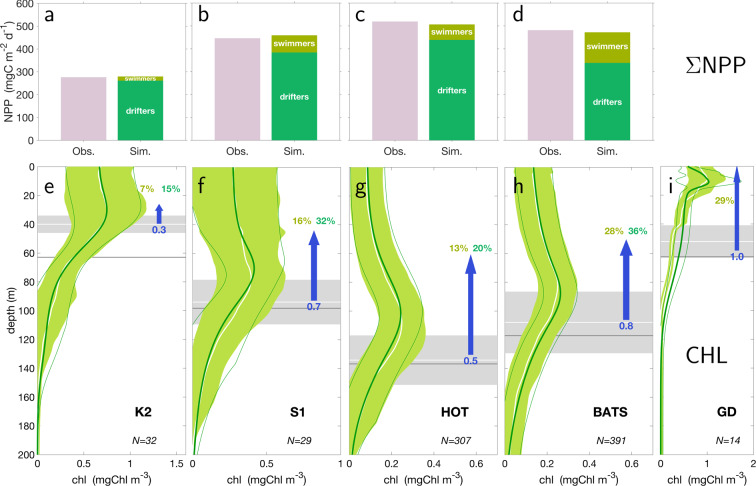
(**a**–**d**) Vertically and temporally integrated net primary production (NPP) rates ($\Sigma$NPP) as observed (pink bars) and calculated by the model (green bars). Green shadings show the contributions to $\Sigma$NPP by actively moving and drifting phytoplankton. (**e**–**i**) Pooled profiles of chlorophyll-a (CHL). Temporal mean (white line) and standard deviation (green area) in observations compared to the mean (thick green line) and standard deviation (thin green line) of simulated profiles. Temporal mean (white line) and standard deviation (grey area) of chemocline depth (${z}_{N}$) are shown for all dates of SCM occurrence. The grey line shows the overall temporal mean. Bold numbers (and proportional widths of blue arrows) are averaged estimates of nutrient release (mmol-N m${}^{-2}$d${}^{-1}$) by active movers. Fractional numbers denote (first) the contribution to $\Sigma$NPP by swimmers, and (second) the fraction of remaining productivity that can be fueled by the upward transport and release of nutrients by swimmers. The vertical position and length of blue arrows corresponds to the average cycling path from ${z}_{C}+\delta z$ to ${z}_{C}-\delta z$ (cf. Fig. 1). From the five marine time-series stations: subarctic West Pacific, K2 (**a**,**e**), subtropic West Pacific, S1 (**b**,**f**), Hawaii Ocean Time-Series, HOT (**c**,**g**), Bermuda Atlantic Time Series, BATS (**d**,**h**), and Gotland Deep, GD (**i**), note that the latter lacks NPP data.

The vertical cycling described herein has the important impact of displacing autotrophs from the chemocline, which favours the formation of subsurface chlorophyll maxima (SCM). According to the parametrization of our model, nutrient uptake declines to 1% of its maximal rate at about 30 m above the chemocline. Up-lifts of SCM by about 20–50 m above the variable chemocline depths (${z}_{N}$) are evident in both observed and reconstructed long-term CHL distributions at the two prominent stations: BATS and HOT in Fig. 2. The SCM peak is therefore generally located in layers where nutrients are strongly limiting. In most cases, ${z}_{N}$ coincides with the depth isoline of low-to-moderate chlorophyll (0.1–0.2 mgCHL m${}^{-3}$) and only very rarely with the SCM depth (i.e. position of the chlorophyll maximum), as for blooms with >0.3 mgCHL m${}^{-3}$ (red colors). Simulated profiles often quantitatively match the observed ones (see also Fig. S2), except that the model does not capture relatively high and low peak CHL concentrations at HOT. Our simulations capture the statistics of the observed CHL distributions for all five marine time-series stations (Fig. 3). This includes precise reconstructions of the average SCM depth and width, and of the temporal variability in CHL profiles. The dependence on the site specific conditions is also captured: average SCM depths range from near-surface (GD) and 30 m (K2) to 110 m (HOT), and average vertical displacements from the chemocline increase from around 10 m at K2, 20 m at S1, 30 m at BATS, to 40 m at HOT and GD. In both the observations and simulations, SCM formation is more likely under relatively shallow temporally averaged ${z}_{N}$, especially when the latter is below 65 m. SCM are less likely with increasing ${z}_{N}$ because although strong mixing during winter deepens the chemocline, it also prevents active movement of phytoplankton.

Given the stark contrast of environmental conditions at these five sites, the reconstructed biological upward transport and release of nitrogen varies less than might be expected: from 0.3 (K2), 0.5 (HOT), 0.7 (S1), 0.8 (BATS) to 1.0 mmol-Nm${}^{-2}$d${}^{-1}$ (GD). The vertical extent of pumping (blue arrows in Fig. 3) roughly corresponds to the width of the SCM at the four oceanic sites. The flux estimates depend on a number of model assumptions such as mortality rates, which are well-constrained by the data given that we have reproduced this large number of CHL profiles by (manually) adjusting relatively few model parameters. For example, the specific mortality rate controls the average height of the SCM, and the tradeoff parameter regulating optimal migration speed affects average SCM position (Fig. S4). Active migrators contribute 7–28% of total calculated $\Sigma$NPP. Their biological N-pumping can explain on average about 25% of the N-demand for production by drifters, from 15% at K2 to 36% at BATS. These direct and indirect contributions combined account for 20–60% of total productivity. This amount can be regarded as a lower bound for sites with surface CHL accumulation and presumably also much higher $\Sigma$NPP, such as GD, where the productivity and CHL concentration peak far above the chemocline. However, at the four oceanic sites, the migration zone does not reach the surface layers where most primary production occurs, as is evident from the moderate contribution of the SCM to $\Sigma$NPP (mostly by swimmers in our model), particularly at the subarctic Pacific site (K2) with its shallow euphotic zones. Nevertheless, our model reproduces the measured values for $\Sigma$NPP, around 300 mg-C m${}^{-2}$d${}^{-1}$ at K2, around 400 at S1, and around 500 at BATS and HOT—all at unprecedented precision.

**Figure 4:**
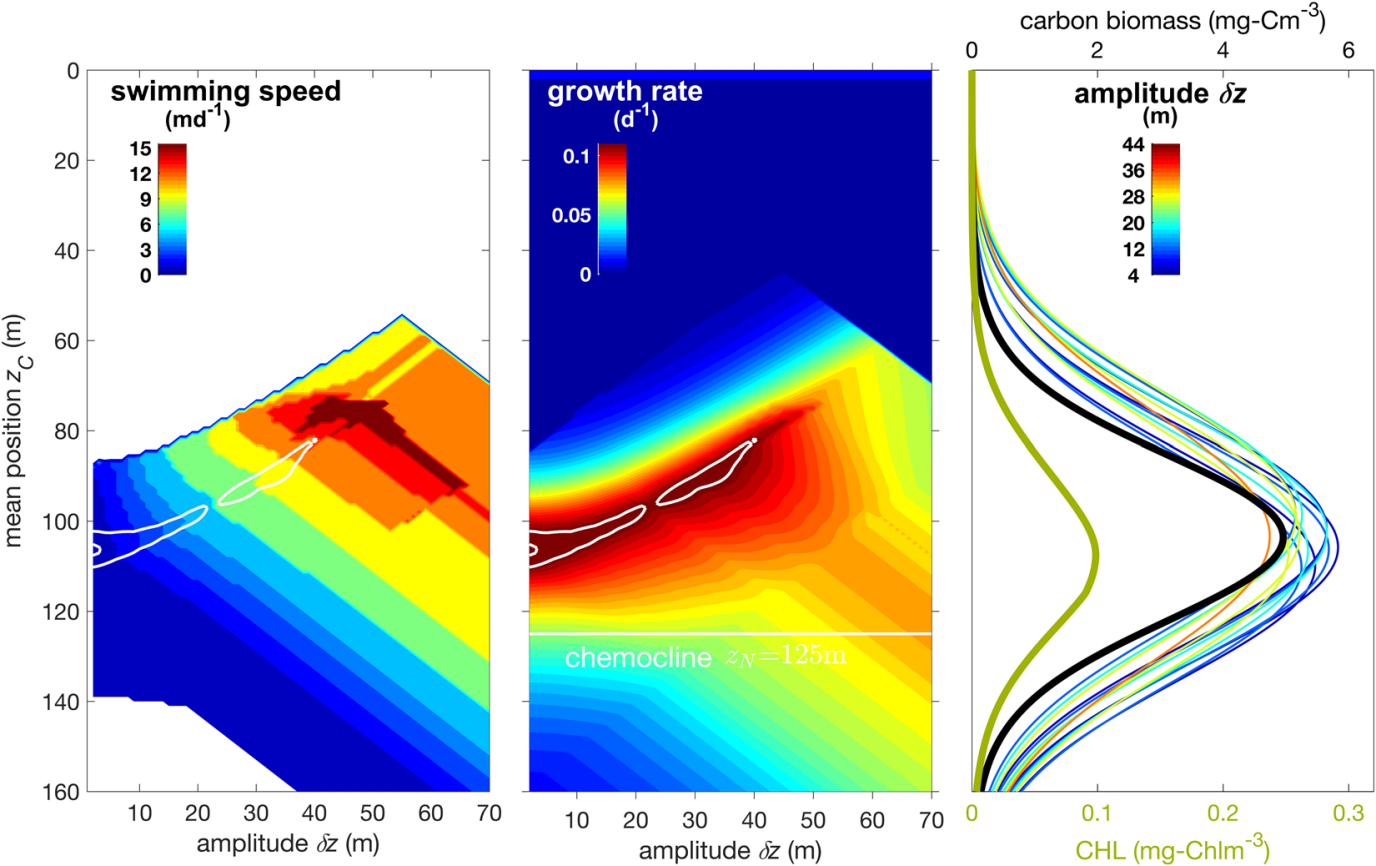
(**a**) Optimal swimming speed depending on mean position ${z}_{C}$ and amplitude $\delta z$. The long white contour line confines solutions with growth rates exceeding 0.95 times the overall maximum, the second contour those beyond 0.99. Environmental conditions are set to chemocline depth ${z}_{N}$=125 m, incident light 180 $\mu$Em${}^{-2}$s${}^{-1}$, and temperature of 20 °C. (**b**) Fitness landscape in terms of two migration traits (${z}_{C}$, $\delta z$). ‘Growth rate’ here refers to net primary production excluding mortality. (**c**) Biomass profiles of (sub)optimal strategies that approximately maximize ’growth rate’ as indicated by the contour line in (**a**,**b**). Line colors denote the corresponding amplitudes $\delta z$. Their sum (here divided by 20, thick black line) gives the carbon profile of the entire subsurface community. The thick olive line shows the corresponding total CHL profile.

**Figure 5:**
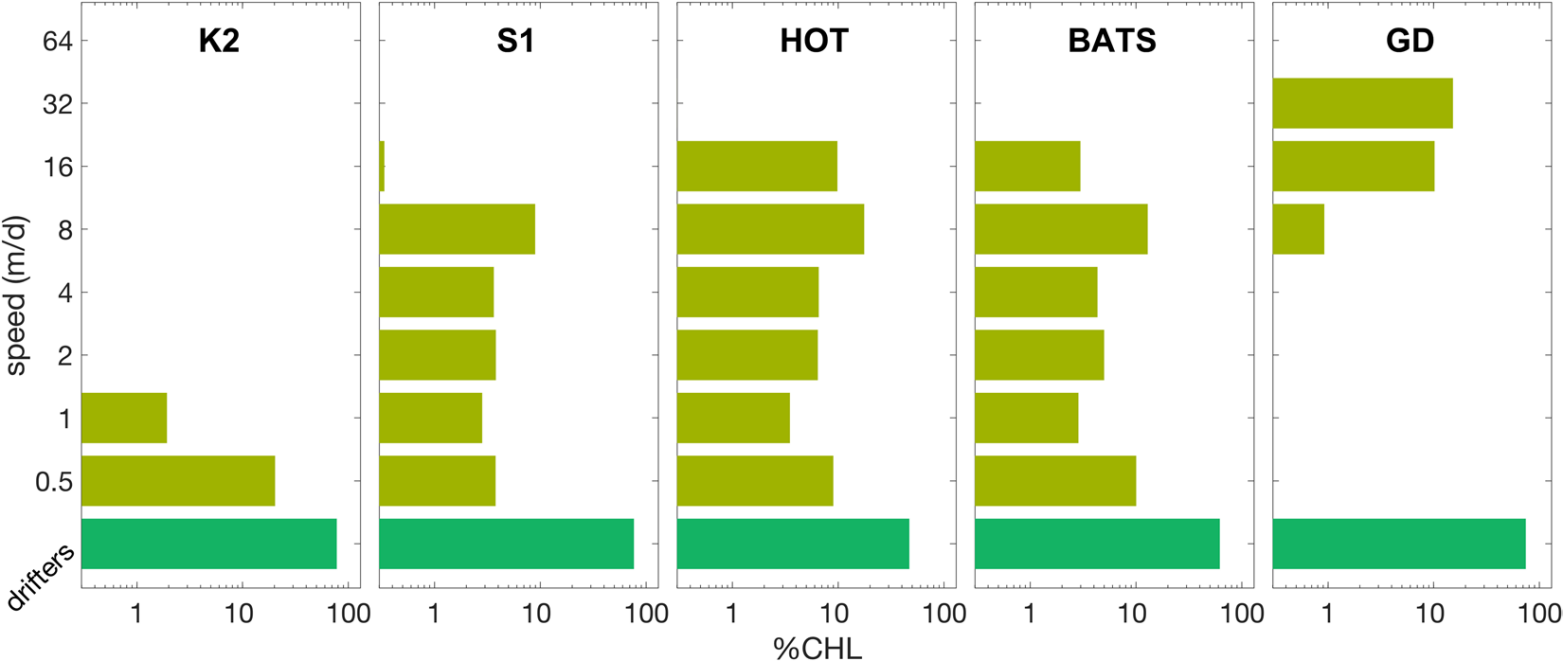
Pigment based community composition (i.e. contribution to simulated total CHL) in terms of mobility for the five marine stations.

Our algorithm selects migration strategies that share very similar growth rates despite a wide range of mean depths and migration widths. A typical solution is displayed in Fig. 4, where a flat, multi-modal fitness landscape arises. Near-optimal strategies with growth rates ranging only from 0.095–0.1 d${}^{-1}$ are predicted for a wide range of half amplitudes from 0–42 m, and corresponding swimming speeds from 0.5–13 md${}^{-1}$. Each strategy is responsible for a uniquely shaped carbon peak that is positioned slightly above the corresponding CHL peak, which happens because CHL:C increases with depth (cf. Fig. S5). The CHL peaks summed together create a broader SCM. To the extent that these distinct strategies are characteristic of different phytoplankton species and groups, neutral fitness suggests a high degree of sustained biodiversity. High biodiversity across all study sites is also evident from the time averaged distribution of near-optimal swimming speeds in Fig. 5. In the model, passive drifters contribute the dominant share of community CHL, from 47% (HOT) to 78% (K2), while mobile phytoplankton of diverse swimming capabilities contribute the remainder. The most skewed distribution emerges at the subarctic site K2, where nearly all swimmers are very slow (0.5 md${}^{-1}$). Distributions tend to be more even at sites with greater ${z}_{N}$, such as BATS, S1 and HOT, where speeds of up to 16 md${}^{-1}$ contribute similarly to CHL. However, strategies with migration speeds of 8 md${}^{-1}$, which are typical of dinoflagellates, contribute a slightly greater fraction of total CHL. A considerable occurrence (25% contribution) of fast migrants with speeds of 16–32 md${}^{-1}$ is only hindcasted at GD. The opitimization scheme did not identify any strategies with speeds faster than that, although no *a priori* limit was imposed.

## Discussion

Given the richness in phytoplankton migration patterns^25^, our understanding of locomotion traits and behaviour remains far from complete. For example, mechanistic triggers of positive and negative phototactic and geotactic movement are barely known across different taxa. Although the ascent velocities of intermediate size diatoms have not been measured, the potential for ascent rates above 1 md${}^{-1}$ is suggested by their positive buoyancy^26^ and capability to persist in a given layer^27,28^. Potentially high adaptive significance of active movement for eukaryotes can also be inferred from the eight (or more) times that phototaxis evolved independently^29^. Autotrophs may share other common regulation mechanisms for vertical movement. Near the surface layers, carbohydrate ballasting facilitated by carbon over-consumption at high photosynthesis rates can result in the efficient transfer of vacuolated forms to the deep. The reversal of this process—consumption of carbohydrates in deeper and darker layers—will generally lower excess densities and therefore increase the probability of ascent.

Occasional up-migration of fast swimming or floating phytoplankton has previously been suggested to act as a nutrient pump from the chemocline towards the surface^2,7^. However, this migration behaviour has been reported for only a few taxa: descending and ascending speeds of several tens of meters per day are realized by large dinoflagellates, colonies of flagellated cells, and non-flagellated floaters regulating their buoyancy such as cyanobacteria and huge diatoms^2,30–32^. Our simulations suggest that dinoflagellates occupy a relevant niche in many areas of the oligotrophic ocean. In addition, fast floaters are the prevailing mobile form at shallow chemoclines in the absence of strong co-limitation because their large storage capacities (for nutrients and carbon) allow efficient sequential usage of light and nutrients over the migratory cycle, greatly enhancing their growth. The observed stoichiometric differences between rapidly sinking and ascending colonies of cyanobacteria constitute some of the best direct evidence for biological nutrient pumping^3^. Nutrient retrieval by diel migration in both freshwater and marine systems has often been linked to phosphorus uptake. Phosphorus was considered the limiting nutrient in our GD application (Baltic Sea), where the calculated pronounced maximum, produced by fast cyclers resting close to the surface, matches both the CHL data and the recurrently observed dominance of N–fixing cyanobacteria at that station. This is essentially the same resource acquisition strategy as the well-known phosphorus retrieval in lakes by short-range and mostly diel migration of cryptomonads and chlorophyceae^30,33^.

Slow movement is much more difficult to trace than diel migration. Vertical swimming direction of dinoflagellates has been reported to follow a bimodal distribution, which indicates either strict upward or downward swimming^34^. Interestingly, bimodality was most pronounced in smaller species compared to larger and faster ones. These insights were made possible through cutting-edge observational methods at the level of single cells. Similar methods, such as cell sorting and single-cell elemental analysis^35,36^, would also be required for the direct verification of our hypothesis, which predicts clear differences in nutrient:C ratios between ascending and descending sub-populations around the central position of the SCM (Fig. 1).

Indirect evidence for active movement in bulk phytoplankton is here provided through extensive model-data comparisons. The agreement of hindcasted and observed NPP confirms the validity of our environmental forcing, comprising temperature, incident light, and chemocline depths ${z}_{N}$ together with the parametrization of light attenuation and phytoplankton physiology. The moderate (10–30%) direct contribution of actively moving phytoplankton to $\Sigma$NPP reflects our generic physiological parametrization. Increasing the light affinity would not only raise this fraction but also enhance growth rates and hence the depletion of intracellular stores, which would in turn limit the migration range (Fig. S4).

The hypothesis of active movement offers a simple and physiologically plausible explanation for the variable displacement of mean SCM positions 10–60 m above the chemocline. Larger displacements, which are most apparent at our study sites HOT and BATS, have also been observed in other regions, such as the Atlantic gyres^37^. In a recent global compilation, significant displacements were found for chemocline depths below 100 m, and displacements reached 80 m for ${z}_{N}$ above 200 m^38^. From the sorting of sites with respect to mean ${z}_{N}$, the displacement remains below 20 m at intermediate ${z}_{N}$ (<100 m), which is in line with independent data sources^25,38^. In contrast, displacements increase to about 50m for either shallow (<60 m) or deep (>100 m) ${z}_{N}$. In the first case (${z}_{N}$ < 60 m), the model predicts fast floating by cells containing gas vacuoles because for them the hydrostatic pressure only becomes critical beyond this depth. Fast floating can be prevented by strong seasonal mixing. The second case (${z}_{N}$ > 100 m) is widespread in the oligotrophic ocean^39^, and offers the greatest benefit for up- and down-migrating cells because of unfavorable light conditions near the chemocline.

Acclimation of the Chl:C ratio enhances visibly the contribution of migrating bulk phytoplankton to the observed CHL profiles. Chl:C increases with depth due to low light and, more importantly, high N quotas of cells having recently visited the chemocline (Fig. S5). As hypothesized previously^25^, Chl:C variations cannot consistently explain SCM formation under the full range of oceanic conditions. The Chl:C ratio can increase pronouncedly for drifters within the chemocline but only smoothly varies above the chemocline.

Realistic variations in optimal migration amplitudes with changing environmental conditions (see Methods for optimality scheme and criteria) underlie the good agreement between observations and hindcasts. Although a wide variety of biogeochemical models have been applied to BATS and HOT, none has yet captured the extensive observed vertical profiles of both CHL and NPP^40^. Models without migration can only reproduce SCM and chemocline position when they are in close proximity, but not when they are separated (Fig. S5) because physical transport cannot operate across vanishing ambient nutrient concentrations. Apart of the biological pumping mechanisms, our model contains only few parameters and many simplifying assumptions. Consequently, the high degree of agreement obtained herein, across very different marine environments, is unexpected. For example, our approach is fully agnostic about ecosystem dynamics, or about taxonomic differences in growth physiology. The major results are not critically affected by variation in the model parameters, as demonstrated for selected cases in Fig. S4. For example, decreasing the specific mortality rate would not only leave the profile shape unaffected but would also increase both the strength of the SCM and the concomitant biological N-pumping rate. Mortality for phytoplankton comprises many variable factors^41,42^, such as infectious diseases, cell lysis, particle aggregation, and herbivores and has here been set to a global density specific constant. Elaboration of any of these factors may further improve the agreement between model results and data.

Besides the substantial portion of NPP attributed to active movers, vertical migration may also indirectly mediate a considerable fraction (15–36%) of the remaining NPP through the pumping of nutrients into the euphotic zone. At all sites, mean N–fluxes fall into the interval of 0.3–1 mmol-Nm${}^{-2}$d${}^{-1}$. These fluxes are 2–5 times larger than typical eddy NO${}_{3}$ injections^6^ and about 30 times larger than calculated for vertically migrating *Rhizosolenia* mats^8^, which can be explained by a rather low standing crop for the latter in comparison to the bulk movement of phytoplankton assumed here. That estimate has recently been raised for a transect in the North-east Pacific to 0.23 mmol-Nm${}^{-2}$d${}^{-1}$ by account for other classically known fast movers, such as huge diatoms or dinoflagellates^6^. Biological pumping may thus constitute an important supply of N in addition to physical pumping by turbulent mixing, particularly during summer when mixing slows down. Cyclic phytoplankton migration has, however, been entirely neglected by state-of-the-art biogeochemical models. This will be difficult to correct because these models resolve phytoplankton exclusively as concentration fields in an Eulerian frame.

Only a few and conceptual concentration-based models have included active movement, by adding a velocity term into the direction of the growth gradient^43,44^. Following the growth gradient is equivalent to an instantaneous optimization of the vertical position, leading to a convergence of cells at a unique optimal location where nutrient and light limitation are balanced. Instantaneous growth optimization can hence explain the formation of thin layers. These models have to assume an unrealistically low nutrient half saturation for growth to sustain phytoplankton at nutrient deprived layers^44^. Their approach differs from our path-dependent optimisation of growth over the entire migration cycle.

Lagrangian models have since long resolved regulation strategies of swimming behaviour depending on external cues and intracellular conditions, which in turn reflect the life history of individual cells^9,45,46^. However, these models simulated cyclic migration and biological nutrient pumping for fast movers, which made it difficult to compare their results with observations that (in general) are available for the entire phytoplankton community. Lagrangian modeling has advanced understanding of the mechanistic origins of vertical migration strategies but it has not yet assessed their impact on community-level metrics, such as bulk vertical distributions and productivity. In contrast, our approach is agnostic about the underlying mechanisms, assuming an optimal migration strategy at the time scale of an individual life-cycle, which has the advantage of being more generic. Its disadvantage is that it requires computationally expensive numerical approaches to identify optimal and sub-optimal strategies. Our novel analytical expression for average growth rate as a function of migration traits and environmental conditions is essential to make this approach feasible for numerical simulations.

Mobility varies with both size and taxonomic group. Migrating cells are typically (large) eukaryotes and non-migrating (small) prokaryotes, with some exceptions given that widespread genera such as diazotrophic *Trichodesmium* may employ both strategies. Our focus on mobility traits nevertheless provides a partial insight into the vertical structuring of community composition. Because CHL:C ratios increase and NPP rates decrease with depth, mobile phytoplankton make a much greater fractional contribution to total CHL than to total productivity. According to our reconstruction, 20–50% of total CHL in the water column belongs to active movers, and a considerable fraction of up to 20% to cyclers with speeds exceeding 4 md${}^{-1}$. This mobility is in line with observations: average swimming velocities of 2–16 md${}^{-1}$^16^ or 18 md${}^{-1}$^47^ have been observed for marine nanoflagellates (with ESD $<$ 9 ${\rm{\mu }}$m). A number of oceanic and shelf communities are at times dominated by motile species^48^, such as in the Baltic Sea^12^, which is consistent with the high share of fast movers reconstructed for GD. Summer blooms of floaters, here large diatoms and the cyanobacterium *Trichodesmium*, were repeatedly recorded in a transect north of HOT^49^. In the oligotrophic Pacific, peak CHL concentrations above 0.15 mg-CHLm${}^{-3}$ consist of more than 30% diatoms and dinoflagellates^50^, which match our modelled fractions for slow movers. At S1, 30–60% of subsurface or SCM CHL falls into the two larger size classes: 3–10 and >10 ${\rm{\mu }}$m (T. Fujiki, pers. comm.). This may translate to a relatively high share of actively moving cells. Also in line with our hypothesis, size distributions along two western Pacific transects (16${}^{\circ }$S–32${}^{\circ }$N) reveal (i) changes in size dominance from smaller (1–6 ${\rm{\mu }}$m) near the surface to larger (6 ${\rm{\mu }}$m) at the SCM, (ii) sporadic near-surface contributions of 10–15% CHL by *Trichodesmium*, and (iii) higher proportions of diatoms and lower proportions of single cell cyanobacteria in the SCM compared to 10 m depth^51^. In contrast, a more recent global transect study^52^ found a slight decrease in diatom abundance with increasing depth, and the lowest fractional CHL contribution from cells larger than 2 ${\rm{\mu }}$m at the SCM. However, the size fractionation method that was used in that study might have erroneously counted the remains of larger cells as part of the smaller size fraction^50^. That global transect study did, however, document increasing nanoflagellate abundance with increasing depth. These qualitative considerations assume that the globally significant photosynthetic prokaryotes such as *Prochlorococcus* lack mobility, while for *Synechococcus* we already referred to evidence of active swimming. Because of the increasing adaptive significance of vertical cycling towards the chemocline, our model broadly predicts an increase in the ratio of eukaryotic:prokaryotic biomass from the surface towards the SCM, which again agrees fully with observations^37,53^.

Slow but active movement in bulk phytoplankton extends the existing view of rapid swimmers and floaters traversing contrasting vertical gradients of resource availability. A different type of evidence for this strategy emerges from Hutchinson’s diversity conundrum. Slow movement can seamlessly support phytoplankton diversity by two mechanisms. First, continuous two-way migration by a wide variety of populations works against vertical zonation into more specialized species-poor communities. Second, the enhancement of phytoplankton growth to a similar degree by a variety of migration strategies creates a multi-dimensional niche in terms of migration amplitude, center, and speed. Our calculations have demonstrated an unexpected flatness in the fitness landscape, which would enable a wider range of strategies, and hence species, to coexist under typical oceanic conditions. The slow growth rates of subsurface phytoplankton further limit potential rates of competitive exclusion, which are proportional to differences in specific growth rates.

## Conclusions

Our model results suggest that a large fraction of phytoplankton, roughly one third in terms of CHL, migrates vertically above the chemocline and that this movement can explain observed vertical profiles of CHL from contrasting regions of the global ocean. The model’s agreement with the data includes the variable displacement of the SCM center above the chemocline, as well as patterns in community composition where larger and more motile species increase with depth as does the eukaryotic:prokaryotic ratio. Active movement has major implications for marine ecosystems and global biogeochemical cycles. Migrating phytoplankton can directly contribute a substantial share of global oceanic NPP (7–28%). Furthermore, their biological pumping of nutrients into the euphotic zone can supply roughly one-third of the demand for total NPP. Remains or exudates of active movers thus may constitute a significant source of regenerated nutrients. Migration also creates additional niches for locomotion strategies with distinct pathways and speeds. Very different strategies can have similar average growth rates. This phenomenon can enhance and sustain diversity in the subsurface phytoplankton community.

We have pieced together trait-based optimality modeling and fragmented knowledge of geotactic and phototactic responses into a new picture of subsurface phytoplankton as *continually active drifters*. In this view, the distribution and biodiversity of phytoplankton, their productivity, and associated biogeochemical fluxes are controlled to a much greater degree by their behaviour than by physical mixing.

## Methods

### Phytoplankton productivity

Phytoplankton biomass is resolved in terms of carbon (C) and nitrogen (N), as expressed by a variable intracellular N:C quota, $Q$. Here we subtract the subsistence demand ${Q}_{0}$ and use the “free” quota $q=Q-{Q}_{0}$. This available resource pool is a major determinant of net photosynthesis rate $P$, as commonly formulated by the Droop–Caperon function, in our notation $q/(q+{Q}_{0})$. $P$ is furthermore controlled by light $I$ according to a piecewise linear Blackman function,

1 $$P=\min \left\{\right.a(q,I)\cdot I,\ {P}_{\max }\left\}\right.\quad with\quad a(q,I)=\alpha \ \frac{q}{q+{Q}_{0}}\ \left(1-\frac{I}{{I}_{\alpha }}\right)$$

with specific light adsorption $\alpha$ and maximum carboxylation rate ${P}_{\max }$. The light affinity term $a(q,I)$ accounts for both the availability of internal resources ($q$) and the flexible partitioning into the light harvesting apparatus^22,54^. Optimal regulation of this partitioning results in the Droop–Caperon dependency of $a(q,I)$, including the effect of down-regulated CHL:C ratio under N depletion^54^. Light affinity $a(q,I)$ also decreases with increasing light, implicitly describing both steady-state photo-acclimation and photo-inhibition.

### Physiological dynamics of migrating cells

The balance equation for the (free) internal nutrient pool $q$ reads

2 $$\frac{dq}{dt}=U-P\cdot (q+{Q}_{0})$$

with nutrient uptake rate $U$. For a *super individual* (i.e., a population of cells sharing the same history and vertical pathway), Eq. 2 must be solved for carbon and nutrient uptake rates that depend on depth and hence on time ($P(t)$, $U(t)$). The solution $q(t)$ describes continuous changes in composition from a Lagrangian perspective, and is here derived in analytical form (Sec. S1). This analytical solution allows computationally efficient simulations, and will facilitate its future implementation within state-of-the-art grid-based models. It traces quantitative changes in the physiology of cells along their migration route within the water column, under the assumption that environmental conditions change in space but not in time during each cycle. Broadly, the path can be subdivided into three phases: (1) downward migration at constant velocity $v$ with quota trajectory ${q}_{+}(T)$, (2) upward movement with ${q}_{-}(t)$, and optionally (3) a resting period at a fixed vertical position. A phytoplankton *super individual* first moves down for a time $T$ ($T$=2$\delta z/v$), such that $q$ increases from $q(t=0)\equiv q(0)$ to ${q}_{+}(T)$ as formulated in Eq. (S4) (see downward branch in Fig. 1), (2) it then turns upward for the same period $T$, with quota decreasing from ${q}_{+}(T)$ to ${q}_{-}(2T)$. If this final value ${q}_{-}(2T)$ exceeds a small threshold, the *super individual* remains in a resting position for a period ${T}_{r}$, changing its quota from ${q}_{-}(2T)$ to ${q}_{r}(2T+{T}_{r})$ as given in Eq. (S1). The latter becomes the starting quota $q(0)$ for the next round. The procedure converges after a few iterations to a limit cycle of $q(t)$ that entails different vertical profiles of $q$ during upward and downward passage (Fig. 1).

### Trade-off between physiology and mobility

Fast swimmers (e.g., dinoflagellates) and fast floaters without gas vacuoles (e.g., huge diatoms) are known to have low growth rates and resource affinities. This decline in physiological performance with motility is here described by a trade-off function for specific light adsorption $\alpha$ and maximum photosynthesis rate ${P}_{\max }$, which both decrease with increasing speed $v$:

3 $$\alpha ={f}_{v}\cdot {\alpha }^{* }\quad {P}_{\max }={f}_{v}\cdot {P}_{\max }^{* }\quad with\quad {f}_{v}=\frac{1+{e}^{-1}}{1+{e}^{(\varepsilon v-{v}^{* })/{v}^{* }}}$$

${v}^{* }$ denotes the speed with highest performance loss and $\varepsilon$ the strength of the non-linear physiological trade-off as controlled by the environment, whereas ${P}_{\max }^{* }$ and ${\alpha }^{* }$ are the reference physiological parameters. All symbols and model coefficients are listed in Table S2. The model parameters were manually changed within realistic limits to obtain overall reasonable results. The effect of changing $\varepsilon$ on the function ${f}_{v}(v)$ is displayed in Fig. S3. $\varepsilon$ should be low for gas vacuolated forms because cyanobacteria only show moderately lower light affinity and maximal photosynthesis rate compared to non- or slow-migratory groups. However, at about 65 m depth, the critical turgor pressure begins to harm or kill cells with gas vacuoles, which prevents fast buoyancy and its regulation^55^. We hence let $\varepsilon$ decrease from one to zero if chemocline depth ${z}_{N}$ < 65 m and if surface nitrate concentration (NO${}_{3}^{0}$) is low so that co-limitation by (for example) iron can be excluded. These two conditions for releasing the mobility–physiology trade-off by decreasing $\varepsilon$ are described by smooth step functions ${f}_{N}$ and ${f}_{z}$ depending on NO${}_{3}^{0}$ (in units mmol-Nm${}^{-3}$) and ${z}_{N}$ (in units m), respectively:

4 $$\varepsilon =\max \{{f}_{z},{f}_{N}\}\quad with\quad {f}_{z}={(1+{e}^{({z}_{N}-65)/2})}^{-1}\quad and\quad {f}_{N}={(1+{e}^{4-{NO}_{3}^{0}})}^{-1}$$

### Optimality in behavioral traits

The idealized migration strategy sketched above can be characterized by four behavioural traits of a phytoplankton *super individual*: migration velocity $v$, mean position ${z}_{C}$ and amplitude $\delta z$ of vertical cycling (in the range ${z}_{C}\pm \delta z$), and the surface resting time ${T}_{r}$. The set of four strategy traits $v$, ${z}_{C}$, $\delta z$, and ${T}_{r}$ together with the input boundary conditions for PAR and chemocline depth determine the hysteresis in the quota (${q}_{\pm }(t,{z}_{C},\delta z,v,{T}_{r})$) as explicitly given in the analytical solutions (Sec. S1). Net photosynthesis rate $P({q}_{\pm }(t,{z}_{C},\delta z,v,{T}_{r}))$ in Eq. (1) is averaged numerically along the cyclic vertical pathway to yield the fitness measure $\bar{P}$. Its dependency on migration strategy $\bar{P}({z}_{C},\delta z,v,{T}_{r})$ enables us to identify the best strategy $({z}_{C}^{* },\delta {z}^{* },{v}^{* },{T}_{r}^{* })$, by using a mixture of discrete loop search and gradient approach on the discretized trait-space: the travel amplitude $\delta z$ is systematically varied from 1–70 m minus the characteristic turbulent mixing length $\delta$ as defined in Sec. S5, so that no long-range residual migration is simulated at strong seasonal mixing where $\delta$ exceeds 50 m. In the remaining cases, the center position ${z}_{C}$ is moved from ${z}_{N}-6$ m to ${z}_{N}$ + 6 m while the upward and downward continuations to more distant depths from the chemocline center are stopped once $\bar{P}$ decreases. If the free quota at the upper turning point exceeds the threshold ${Q}_{0}$, then a resting period is added (Sec. S2). For each pair of ${z}_{C}$ and $\delta z$, a discrete set of swimming/floating speed $v={2}^{n}$ md${}^{-1}$ with $n$=$-1,\ldots 6$ is evaluated with respect to maximal $\bar{P}$.

### Vertical distribution of mobile cells

The strategy traits $v$, ${z}_{C}$, $\delta z$ determine the biomass distribution function $\varphi (z)$ of vertically moving cells. This function should be rectangular given our assumption of constant traveling speed. However, $\varphi (z)$ is smoothed as cells are dispersed randomly by turbulence, which we assume to exceed microscale displacement by non-directed locomotion. The length scale of total dispersal ($\delta {z}_{di\mathrm{ff}}$) can be linked to the turbulent diffusion length $\delta$ (normalised to one day, see Sec. S5) and one-way travel time $T=2\delta z{v}^{-1}$, thus $\delta {z}_{di\mathrm{ff}}=(\delta +4m)\cdot \sqrt{2\delta z\ {v}^{-1}}$, where a small offset is added to account for bio-diffusion due to (for example) non-directed swimming. By taking the rectangular distribution defined by the mean position ${z}_{C}$ and the migration amplitude 2$\delta z$ as initial condition, the physical reshaping of $\phi (z)$ follows from the analytical solution of the one-dimensional diffusion problem at time $T$, which is the sum of two error functions:

5 $$\phi (z)=\frac{1}{4\delta z}\left[erf\left(\frac{z-{z}_{C}+\delta z}{\delta {z}_{di\mathrm{ff}}}\right)+erf\left(\frac{{z}_{C}-z-\delta z}{\delta {z}_{di\mathrm{ff}}}\right)\right]$$

By construction, the depth integral of the probability density function $\varphi (z)$ equals one. In the relatively rare case of a non-zero resting period (${T}_{r}$ > 0, see above), the density function $\varphi (z)$ is extended to include near-surface biomass accumulation (Sec. S2).

In addition to physical mixing, vertical biomass profiles of the mobile phytoplankton are smoothed by a release of the strict optimality assumption. Different migration behaviours, being either close or distant to each other, may result in very similar growth performances, for which we infer low competitive exclusion (Fig. 4). We thus collect all strategies with nearly optimal performance, ${\bar{P}}_{i}$ = $\bar{P}({z}_{C,i},\delta {z}_{i}) > 0.95\cdot {\max }_{i}{\bar{P}}_{i}$. Their distribution functions ${\varphi }_{i}(z)$ are then summed, weighed with their relative fitness ${w}_{i}$ = ${\bar{P}}_{i}$/${\sum }_{j}{\bar{P}}_{j}$, to give the total vertical distribution function ${\varphi }_{tot}(z)$ of actively moving cells, ${\varphi }_{tot}(z)={\sum }_{i}{w}_{i}{\phi }_{i}(z)$.

### Density dependent mortality and biomass profiles

Our model differentiates between two fractions of the phytoplankton community: vertically migrating cells and non-migrating cells. The depth dependent C concentration of actively moving phytoplankton is denoted by ${C}_{a}(z)$ = ${\varphi }_{tot}(z)\widetilde{C}$ with cumulative concentration $\widetilde{C}$, the C concentration of passively drifting or “immobile” phytoplankton by ${C}_{p}(z)$. The “immobile” and “mobile” phytoplankton interact indirectly through light attenuation (Eq. (S10)) and density dependent mortality. Causes of mortality can include viral diseases, aggregate formation with detritus particles or live cells, and zooplankton grazing at low prey concentration. These factors are collected into the relative mortality rate $M$ that is a function of temperature (${f}_{T,m}$) and total biomass concentration,

6 $$M=m\ {f}_{T,m}\cdot ({C}_{p}+{C}_{a})$$

with the specific mortality coefficient $m$. Assuming steady-state in biomass dynamics, thus vanishing net growth at equal loss and production, $P-M=0$, and approximating ${C}_{a}\approx \widetilde{C}/2\delta z$, we obtain the cumulative concentration $\widetilde{C}$:

7 $$\widetilde{C}=2\delta z\cdot \left(\frac{P}{m{f}_{T,m}}-{C}_{p}\right)$$

Taken together, the vertical shape of the biomass profile of active movers ${C}_{a}(z)$ reflects the optimal migration traits through ${\varphi }_{tot}(z)$. Absolute values of ${C}_{a}(z)$ are determined by the productivity ($P$) and the presence of passive drifters (${C}_{p}$) as given by $\widetilde{C}(P,{C}_{p})$. While the chlorophyll profile for the mobile fraction ${Chl}_{a}(z)$ follows from multiplication of ${C}_{a}(z)$ with the depth dependent CHL:C ratio, the profile ${Chl}_{p}(z)$ of the passive drifters is directly estimated in Eq. (S9), which is then transformed to ${C}_{p}(z)$ through division by Chl:C. The latter in turn increases with free N-quota $q$ and decreases with light $I$ (cf. $a(q,I)$ in Eq. (1)) according to

8 $$Chl:C={c}^{* }+q\ \theta \cdot (1-I/{I}_{\alpha })$$

The sum of ${Chl}_{a}(z)$ and ${Chl}_{p}(z)$ gives the total chlorophyll profile that is compared to the field data.

### Cumulative productivity and nutrient pumping

Similar to the calculations for CHL profiles, multiplying biomass profiles ${C}_{a}(z)$ with the production rates averaged along the cyclic vertical pathway ($\bar{P}$) yields the net primary productivity (NPP) of the mobile fraction. For the passively drifting fraction, we assume a linear decrease in the (free) nutrient-to-carbon quota ${q}_{p}(z)$ from the chemocline to the top-most position (cf. Fig. 1, ${q}_{p}(z)$ = ${Q}_{0}/10+({q}_{\max }-{Q}_{0}/10)\ast z/{z}_{N}$), which affects both photosynthesis in Eq. (1) and Chl:C ratio in Eq. (8). We assume implicitly that this fraction takes up mostly regenerated nutrients, with additional sources from atmospheric deposition and nitrogen fixation. Note that passive drifters share the same growth parameters with the mobile population, as end-members with $v$ = 0 of the mobility–physiology trade-off Eq. (3). The two NPP contributions are summed over the entire water column, which results in integrated net primary production rates ($\Sigma$NPP).

The amount of nutrients pumped into the euphotic zone and released by vertically migrating cells is estimated as the product of (1) the average N:C ratio ($\bar{Q}$) above the chemocline ($z < {z}_{N}$) of the migration cycle, (2) the vertically integrated carbon concentration ($\Sigma {C}_{a}$), and (3) the relative mortality rate ($M$).

### Data integration and boundary conditions

Publicly available data for vertical profiles of chlorophyll, nutrients, and primary production were obtained from the following five time-series observation sites (also listed in Table S1). The most extensive datasets are from two long-term subtropical sites. Substantial seasonality in nutrient levels and production are observed at the more variable Bermuda Atlantic Time-Series (BATS, http://bats.bios.edu), whereas the Hawaii Ocean Time-series (HOT, http://hahana.soest.hawaii.edu/hot/hot-dogs/) is more calm and oligotrophic, with a persistent deep chlorophyll layer. The less extensive data sets available from the two NW Pacific sites maintained by JAMSTEC nevertheless resolve contrasting environments, with strong seasonality, high nutrient concentrations, and relatively low light at subarctic station K2, in contrast to somewhat weaker seasonality, persistently low nutrient concentrations, and stronger light at subtropical station S1 (http://ebcrpa.jamstec.go.jp/k2s1/en). Phosphate and chlorophyll data for the Gotland Deep (GD) site from 2006–2008 originate from the Leibniz Institute for Baltic Sea Research Warnemünde, Germany (IOW) and can be downloaded from https://odin2.io-warnemuende.de/data. For all stations, temperature data were averaged over the upper 100 m.

We estimate chemocline depth ${z}_{N}$ using the observed profile data for ${NO}_{3}$ – or ${PO}_{4}$ for Gotland Deep (GD). We first identify the depth at which the standard deviation in nutrient concentration relative to the surface exceeds a threshold of 0.1 mmol-m${}^{-3}$ (multiplying by Redfield N:P ratio 15 for ${PO}_{4}$ at GD), and then add 20 m to estimate the chemocline center position.

Short wave radiation data for the five stations were obtained from the Japan Meteorological Agency JRA-55 re-analysis, based on atmospheric data assimilation covering years 1958–2012 (http://jra.kishou.go.jp/JRA-55/index_en.html#jra-55). Reconstruction of environmental and boundary conditions based on the station data is described in the Supplementary Information: the base CHL profile ${Chl}_{p}(z)$ in Sec. S3, light attenuation in Sec. S4, turbulent mixing in Sec. S5, and temperature dependencies in Sec. S6.

## Supplementary information

Supplementary Information.
