# Author Correction: Vertical migration by bulk phytoplankton sustains biodiversity and nutrient input to the surface ocean

**DOI:** 10.1038/s41598-021-88994-y

**Published:** 2021-04-28

**Authors:** Kai Wirtz, S. Lan Smith

**Affiliations:** 1 Institute of Coastal Research, Helmholtz Centre Geesthacht, Geesthacht, Germany; 2 Earth Surface System Research Center, Research Institute for Global Change, JAMSTEC, Yokosuka, Japan

Correction to: *Scientific Reports* 10.1038/s41598-020-57890-2, published online 24 January 2020

The Supplementary Information published with this Article contains errors, where a number of the hyperlinks inside the document do not function.

The correct Supplementary Information file is provided below.

## Supplementary Information

Supplementary Information.

