# Supplementary material for: Author Correction: Vertical migration by bulk phytoplankton sustains biodiversity and nutrient input to the surface ocean
Source: Sci Rep. 2021 Apr 28;11:9486. doi: 10.1038/s41598-021-88994-y (PMC8081721; doi:10.1038/s41598-021-88994-y)
Supplement: Supplementary file 1 — Supplementary Information. [file 41598_2021_88994_MOESM1_ESM.pdf]

Supporting Online Material

”Vertical migration by bulk phytoplankton sustains biodiversity and nutrient input to the surface ocean”

Kai Wirtz[1,*] **and S. Lan Smith**[2]

[1]Institute of Coastal Research, Helmholtz Centre Geesthacht, Geesthacht, Germany. kai.wirtz@hzg.de
[2]Earth SURFACE System Research Center, Research Institute for Global Change, JAMSTEC, Yokosuka, Japan

ABSTRACT

S1 Solutions of the quota–balance equation

Our approaches requires evaluating optimal migration strategies using a numerically fast calculation of nutrient quota dynamics for migrating cells. Fast computation will also facilitate integration into state-of-the-art Eulerian models in the future. We assume that during each migration cycle, environmental conditions vary with depth but not with time, and calculate the variable nutrient quota and resulting average growth rate during that cycle based on each observed nutrient profile and corresponding solar radiation data from a re-analysis product. In the following, we derive analytical solutions of the nutrient quota for three cases: (1) an immobile cell at fixed depth, (2) a migrating cell at (2a) light limitation, and (2b) light saturation

(1) If the cell resides at a fixed depth, average light I, production rate P and nutrient uptake rate U are constant. Time variations at the right hand side of Eq. (2) only originate from a linear dependency on free quota q, allowing the simple solution

$$q_r(t) = q_1 + q_2\,\mathrm{e}^{-pt} \tag{S1}$$

The coefficients q_1, q_2 and p are set according to whether the cell rests in the light-limited part of the water column (where $a(q,I)\,I < 1$), or in the sun-lit surface layer. Under light limitation, quota dependency includes the adsorption–Droop term $q/q+Q_0$, leading to $U - pq$ at the right hand side of Eq. (2) with p=$P_{\max}\alpha(1-I/I_\alpha)I$ and q_1=U/p. From the initial condition $q_r(t=0)$=$q(0)$, we have q_2=$q(0)-q_1$. Under light saturation, the subsistence quota contribution in $(q+Q_0)P_{\max}$ can be shifted to the uptake term such that the coefficients read p=$P_{\max}$, q_1=$(U-Q_0p)/p$, and again q_2=$q(0)-q_1$. However, the resulting exponential depletion of internal nutrient stores will quickly lower light affinity to the point that light becomes limiting ($a(q,I)\,I < 1$). Without refurnishing supplies, thus U=0, $q_1 \leq 0$, and regardless of the degree of light limitation, internal stores are depleted proportional to the specific production rate (p).

The exponential decrease of $q_r(t)$ in Eq. (S1) reflects a resource depletion acting through time and proportional to the current value of $q_r(t)$ itself. Intracellular depletion of quota is faster at high q, when both production and nutrient demand are high.

(2) As cells move vertically, rates of both net primary production P and nutrient uptake U in Eq. (1)–(2) vary, and our approach requires an explicit formulation for their dependence on ambient conditions. Nutrient uptake rate U depends linearly on depth-dependent ambient nutrient concentration, because we omitted an explicit account of uptake saturation and down-regulation at high internal nutrient stores. Instead, we imposed the boundary condition $q < q_{\max}$, which suffices to capture spatio-temporal trends in q, because our approach neglects temporal changes in the ambient nutrient pool.

The dependence of phytoplankton physiology on migration history demands a shift in the coordinate system, from grid-based (Eulerian) to individual-based (Lagrangian). In this Lagrangian view, ambient conditions such as nutrient availability and light vary along the vertical pathways of individual cells. Nutrient uptake U then depends exponentially on vertical position z, $U{=}u^{*}\mathrm{e}^{k_{\mathrm{N}}\cdot(z-z_{\mathrm{N}})}$, in accordance with the often observed steep rise in nutrient concentration around the chemocline position z_{N} (see Fig. S2). At the chemocline center, U is by construction given by a global constant u^{*}. Similarly, light availability, I, varies for a cell migrating within the chemocline, but in the opposite direction: $I{=}I_N\mathrm{e}^{-k_I(z-z_{\mathrm{N}})}$ with date– and site–dependent irradiance I_N at the chemocline center ($z{=}z_{\mathrm{N}}$, Sec. S4) and k_I described in Eq. (S12) .

For a compact description of variations in both chemical and light conditions we introduce the variable $\zeta(t){=}\mathrm{e}^{-k_I(z(t)-z_{\mathrm{N}})}$, which represents the experienced light level relative to the level at the chemocline center z_{N} (i.e. ζ equals one at $z(t){=}z_{\mathrm{N}}$). As shown in Fig. 1 and Fig. S2, the slope of the chemocline is much steeper than that of the photocline. The relation $k_{\mathrm{N}}{=}3k_I$ provides a good fit to the NO_3 data and enables to use the position variable ζ also for describing variations in the uptake rate ($U{=}u^{*}\,\zeta^{-3}$, with u^{*} denoting the nutrient uptake rate at the central chemocline). The new variable ζ not only represents the vertical position, but can also be substituted for time t for a cell assumed to move at constant vertical speed v. This movement can be either downward, $\mathrm{d}z/\mathrm{d}t{=}v$, or upward, $\mathrm{d}z/\mathrm{d}t{=}-v$, so that

$$\frac{\mathrm{d}\zeta}{\mathrm{d}t}=\frac{\partial\zeta}{\partial z}\frac{\mathrm{d}z}{\mathrm{d}t}=\mp v k_I\zeta \tag{S2}$$

(2a) In the light-limited part of the water column (where $a(q,I)\,I<1$), the balance equation can be transformed into a differential equation in the relative light level ζ using Eq. (S2) :

$$\frac{\mathrm{d}q}{\mathrm{d}t}=u^{*}\,\zeta^{-3}-p^{*}\,\zeta q \qquad\longrightarrow\qquad \frac{\mathrm{d}q}{\mathrm{d}\zeta}=p_{\pm}q-u_{\pm}\,\zeta^{-4} \tag{S3}$$

The rescaled photosynthesis rate, $p_{\pm}{=}\pm p^{*}/k_I v$, is dimensionless, and can be interpreted as potential production relative to a typical rate of change in light availability; the rescaled uptake $u_{\pm}{=}\pm u^{*}/k_I v$ has the units of a nutrient quota. Eq. (S3) can be solved analytically,

$$q_{\pm}(\zeta)=q_{2\pm}\,\mathrm{e}^{p_{\pm}\zeta}+\frac{u_{\pm}}{6}\left(p^{3}\,\mathrm{e}^{p_{\pm}\zeta}\mathrm{Ei}(-p_{\pm}\zeta)+\frac{p_{\pm}^{2}}{\zeta}-\frac{p_{\pm}}{\zeta^{2}}+\frac{2}{\zeta^{3}}\right) \tag{S4}$$

where the integration constant $q_{2\pm}$ is determined by the initial condition $q_{\pm}(\zeta(0)){=}q(0)$.

The special function called exponential integral $\mathrm{Ei}(\zeta)$ can be well approximated by series expansions, and is made available within many numerical packages. Unlike case (1) for resting cells, vertical migration leads to great variations in the specific exponential argument of Eq. (S4) over time. If the swimming is downward directed ($p_{\pm}{=}{+}p^{*}/k_I v$), the positive specific argument $p_{\pm}$ describes an accelerating rise in internal nutrient quota during the passage as photosynthesis steadily declines while ambient nutrient levels increase.

On the contrary, for upward swimming ($p_{\pm}{=}{-}p^{*}/k_I v$), the specific rate becomes negative and this depletion rate increases with light availability ζ towards the surface. This change of ζ in turn depends on the vertical swimming speed v and light extinction coefficient k_I defined in Eq. (S10) . Although both factors negatively affect the specific coefficient in the exponential function, their net effect on the argument in the exponential function is positive, because the two factors enhance differences in ζ (or $\zeta(t)-\zeta(0)$) along the trajectory of the cell.

(2b) When a cell enters the surface layer where light is saturating, $a_I Iq/(q+Q_0)>1$, the balance equation Eq. (2) reads

$$\frac{\mathrm{d}q}{\mathrm{d}t}=u^{*}\,\zeta^{-3}-P_m\,(q+Q_0) \tag{S5}$$

with solution $q_{s\pm}(\zeta(t))$ in analogy to the light limited solution Eq. (S4)

$$q_{s\pm}(t)=\frac{u^{*}\,\zeta^{-3}(t)}{P_m\pm k_N v}-Q_0+q_{2\pm}\,\mathrm{e}^{-P_m t} \tag{S6}$$

Again, the coefficient $q_{2\pm}$ makes sure that $q_{s\pm}(0)$ matches the initial value of the intracellular free quota: $q_{2\pm}=q(0)+Q_0-u^*\zeta^{-3}(0)/(P_m \pm k_N v)$.

S2 Near-surface resting

Our algorithm for simplicity includes the scenario that cells rest near the surface but not at or below the deeper chemocline because at very high nutrient concentration and virtually no light, stopping has practically the same effect as extending migration deeper. A near-surface resting period is added, if the free quota at the upper turning point ($z=z_C-\delta z$) exceeds the threshold Q_0. Resting time T_r is then estimated using Eq. (S1) as the time needed for the free quota q to reach the threshold Q_0, thus $T_r=\log(q/2Q_0)/p$, with rescaled photosynthesis rate p introduced in Eq. (S1) .

Near-surface resting modifies the biomass distribution function of active movers, $\phi(z)$, formulated in Eq. (5) . The algorithm adds a contribution $\phi_r(z)$, which like $\phi(z)$ arises from the solution of the one-dimensional diffusion equation. Dirac-type initial conditions at $z=z_C-\delta z$ generate a Gaussian function, with diffusion length δ (Sec. S5)

$$\phi_r(z) = \frac{1}{\sqrt{2\pi}\delta}\ e^{-\dfrac{(z-z_C+\delta z)^2}{2\delta^2}} \tag{S7}$$

The ratio of T_r over total migration time, $w_r=T_r/(2T+T_r)$, becomes the relative weight of the resting density ϕ_r when adding to $\phi(z)$

$$\phi(z) \to w_r\phi_r(z) + (1-w_r)\phi(z) \tag{S8}$$

S3 Base CHL profile

Complementarity between observed nitrate and chlorophyll-a profiles is often apparent in the time-series data at all five marine stations in cases of absent subsurface chlorophyll maximum (SCM). This finding is generalized to estimate the CHL concentration of the passively drifting or ”immobile” population $\mathrm{Chl_p}$ constrained by the observed surface chlorophyll concentration $\mathrm{Chl_0}$. If typical mixing rates by far exceed growth and photo-acclimation rates, both biomass and pigment concentration will be constant throughout the inhabited surface zone.

$$\mathrm{Chl_p}(z) = \mathrm{Chl_0}\,\frac{1+e^{-k_\mathrm{N} z'_\mathrm{N}}}{1+e^{-k_\mathrm{N}\cdot(z'_\mathrm{N}-z)}} \tag{S9}$$

The mean chemocline slope k_N is set to three times the mean light attenuation coefficient (Sec. S1). The CHL penetration depth z'_N is slightly extended into the chemocline by 10m and in relation to temperature by adding 1.6 m per degree C decrease in temperature. In addition, the observed surface value $\mathrm{Chl_0}$ is reduced by the factor f_z in Eq. (4) at shallow chemocline depth, suggesting a contribution of the SCM to surface phytoplankton. However, this modification only applies at in the absence of co-limitation, hence for very small f_N in Eq. (4) and within the (summer) low turbulence period (Sec. S5).

Under co-limitation ($\mathrm{NO_3^0} > 0.3\mathrm{mmol\text{-}Nm^{-3}}$) and high surface CHL ($>0.3\mathrm{mg\text{-}CHLm^{-3}}$), the Chl:C profile of drifters is smoothed by raising the lower threshold c^* in Eq. (8) by a factor of two.

S4 Light attenuation

Depth dependent light availability, $I(z)$, is proportional to the surface incident irradiation I_0,

$$I(z) = I_0\ e^{-\int_0^z \left(k_0 + k_\mathrm{chl}\mathrm{Chl}(z')\right) dz'} \qquad \text{with} \quad \mathrm{Chl} = \mathrm{Chl_p} + \mathrm{Chl_a} \tag{S10}$$

and accounts for attenuation by self-shading of the phytoplankton population determined by the attenuation coefficient k_chl and the sum of pigments in passive drifters ($\mathrm{Chl_p}$, see Eq. (S9)) and in active movers ($\mathrm{Chl_a}$).

Other optically active constituents such as colored dissolved organic matter (CDOM) are here subsumed with coefficient k_0. The seasonal rise in attenuation[1] is expressed in terms of the time-variable turbulent mixing length δ (Sec. S5): both specific coefficients $k_{\rm chl}$ and k_0 are multiplied with a factor $\max\{1, \delta/30m\}$, thus increase during winterly mixing.

The concentrations of biological constituents such as CDOM vary not only in time but also between sites. We suggest that the concentrations correlate with the long-term average of surface chlorophyll $\overline{\rm Chl}_0$ (in units mg-CHLm^{-3}), from which we formulate site-specific differences in water attenuation (in units m^{-1}):

$$k_0 = 0.034 + 0.1 \cdot \overline{\rm Chl}_0 \quad \text{(S11)}$$

Note that the dependency of k_0 on average CHL levels can be interpreted as an extra contribution of the CHL-specific attenuation coefficient $k_{\rm chl}$. If $k_{\rm chl}$ is retrieved from correlation analysis, the model value should thus be slightly below the empirical estimate. From the wide scatter in $k_{\rm chl}$ from around 0.01–0.08 mg-CHL^{-1} m^2[2], our setting $k_{\rm chl}$=0.04 mg-CHL^{-1} m^2 appears reasonable, especially in light of its seasonal enhancement.

Total light attenuation k_I near the chemocline can be approximated

$$k_I = k_0 + k_{\rm chl} \cdot \left(\text{Chl : C} \cdot \frac{\widetilde{C}}{2\delta z} + \text{Chl}_0 \right) \quad \text{(S12)}$$

where the Chl:C ratio is calculated from Eq. (8) inserting q=0.4$q_{\max}$ and I=I_N=$I_0 \cdot \exp(-k_I z_{\rm N})$.

S5 Seasonality in turbulent mixing

In our study, the turbulent mixing length δ describes the vertical dispersal range during one day. δ is approximated using long-term satellite-derived data for sea surface temperature (SST, or T) for each station obtained from www.esa-sst-cci.org.

The amplitude of seasonal mixing is suggested to reflect the SST seasonality of each site. The seasonal amplitude should be low if the annual standard deviation in SST σ_T is below a threshold, and large for high σ_T, with a smooth transition. Summer stratification occurs in our simple approximation depending on the current and positive SST deviation from the temporal mean $\overline{T}$, thus T'=$\max(T - \overline{T}, 0)$, and furthermore on the monthly change in SST, if positive, thus δT_+=$\max(T(t+15d) - T(t-15d, 0)$. Together, mixing length (m) depends on the SST statistics (^{o}C)

$$\delta = 2.4\text{m} + \frac{85\text{m}}{1 + \text{e}^{3 \cdot (3.4 - \sigma_T)}} \cdot \frac{1}{1 + \text{e}^{T'_+ + 3\delta T_+ - 2}} \quad \text{(S13)}$$

Coefficients were estimated based on physical model results for S1 and HOT[3]. Near tropical sites such as HOT with low seasonality only display a moderate increase in δ during the mixing season, while sites at higher latitudes such as GD and K2 exhibit strong winter mixing as shown in Fig. S1.

In case of strong mixing (δ>8m), growth conditions of drifting phytoplankton are adjusted to growth and quota values that are averaged for each layer z from $z - \delta$ to $z + \delta$. For example, light $I(z)$ is modified to $I(z) \cdot \left(\text{e}^{-k_I(z-\delta)} - \text{e}^{-k_I(z+\delta)} \right) / 2k_I\delta$.

S6 Temperature dependencies

Temperature dependency f_T of maximum photosynthesis $P_{\max}$ (Eq. (1)) and nutrient uptake rate U (Eq. (2)) is assumed to follow the Q_{10} rule, with $Q10 \approx$1.8[4].

$$f_{T(m)} = Q10_{(m)}^{(T-20^o)/10^o} \quad \text{(S14)}$$

Mortality related processes (see M in Eq. (6)) are here assumed to predominantly originate from grazing, which typically is more temperature sensitive (higher $Q10$) (see Tab. S2).

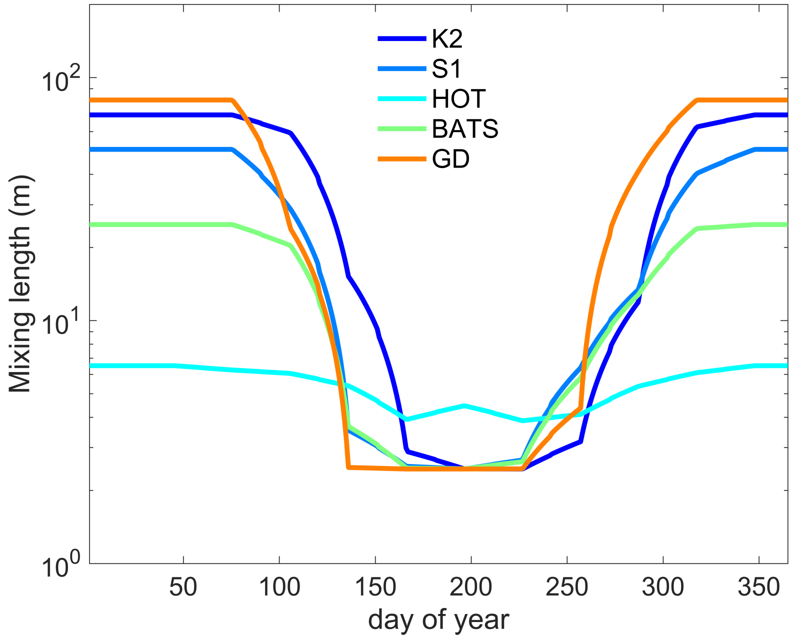

Figure S1. Annual cycles in mixing length δ reconstructed using SST data for the five time-series stations.

Table S1. Time series stations with the number of CHL profiles used in this study (N), average chemocline depth $\bar{z}_N$, and average temperature $\overline{T}$.

	Station	coord.	N	$\bar{z}_N$ (m)	$\overline{T}$ (^{o}C)
K2	Subarctic West Pacific	160W 47N	33	82	4.3
S1	Subtropic West Pacific	145W 30N	31	95	21.1
HOT	Hawaii Ocean Time-Series	-157.8W 22.8N	304	138	25.0
BATS	Bermuda Atlantic Time Series	-64.1W 31.8N	399	113	21.5
GD	Gotland Deep	20.1W 57.3N	14	62	5.8

Table S2. Parameter values and symbols used in the model, including optimized trait variables (TV).
*The seemingly low value for P_{max} exceeds the values recently reported for two dominant groups in the South Atlantic[5] and corresponds to a global compilation[6] assuming a CHL:C ratio of 0.016 gCHLgC^{-1}.

Symbol	Description	Value	Unit
a	Light affinity		m^{2}Wd^{-1}
α	Specific light affinity	0.011	m^{2}Wd^{-1}
c^*	Base CHL:C ratio	0.01	g-CHLg-C^{-1}
C_a	Concentration of "mobile" phytoplankton		mg-Cm^{-3}
C_p	Conc. "passive drifters"		mg-Cm^{-3}
$\widetilde{C}$	Cumulative conc. "mobile"	Eq. (7)	mg-Cm^{-2}
Chl:C	Chlorophyll:C ratio	Eq. (8)	g-CHLg-C^{-1}
$\mathrm{Chl_a}$	CHL conc. "mobiles"		mg-CHLm^{-3}
$\mathrm{Chl_p}$	CHL conc. "drifters"	Eq. (S9)	mg-CHLm^{-3}
$\mathrm{Chl_0}$	Surface CHL conc.		mg-CHLm^{-3}
δz	Vertical migration amplitude	(TV)	m
ε	Strength of physiological trade-off f_v	Eq. (4)	
f_v	Performance reduction at high v	Eq. (3)	
f_T	Physiological temperature dependency	Eq. (S14)	
$f_{T,m}$	Ecological temperature dependency	Eq. (S14)	
I	Shortwave radiation	Eq. (S10)	Wm^{-2}
I_0	Incident light at surface		Wm^{-2}
I_N	Light at chemocline center	Eq. (S10) (z=z_N)	Wm^{-2}
I_α	Photo-sensitivity	70+3I_N	Wm^{-2}
k_{chl}	CHL-specific light attenuation	0.04	mg-CHL^{-1} m^2
k_0	Light attenuation in water	Eq. (S11)	m^{-1}
k_I	Total light attenuation	Eq. (S12)	m^{-1}
M	Density dependent mortality	Eq. (6)	d^{-1}
m	Specific density dependent mortality	0.0043	mg-C^{-1}m^{3}d^{-1}
P_{max}	Maximum photosynthesis rate	0.7*	d^{-1}
P	Specific photosynthesis rate		d^{-1}
ϕ	Vertical biomass density of movers	Eq. (5)	m^{-1}
ϕ_r	Vertical biomass density of resting movers	Eq. (S7)	m^{-1}
Q_0	Subsistence quota	0.03	mol-N (mol-C)$^{-1}$
Q	Intracellular nutrient quota		mol-N (mol-C)$^{-1}$
q	Free intracellular quota ($Q-Q_0$)		mol-N (mol-C)$^{-1}$
q_{max}	Maximum free quota	0.2	mol-N (mol-C)$^{-1}$
$Q10$	Q10 of autotrophic processes	1.8	
$Q10_m$	Q10 of heterotrophic processes	2.5	
T_r	Resting time near surface	(TV)	d
θ	CHL:N ratio	0.1	g-CHLmol-N^{-1}mol-Cg-C^{-1}
U	Nutrient uptake rate		mol-N(mol-C)$^{-1}$d^{-1}
u^*	Nutrient uptake rate at chemocline	0.2	mol-N(mol-C)$^{-1}$d^{-1}
v	Swimming/floating speed	(TV)	md^{-1}
v^*	Critical speed in physiological trade-off	10	md^{-1}
z_N	Chemocline center position		m
$z(t)$	Concurrent depth of phytoplankton cell		m
z_C	Average vertical position	(TV)	m

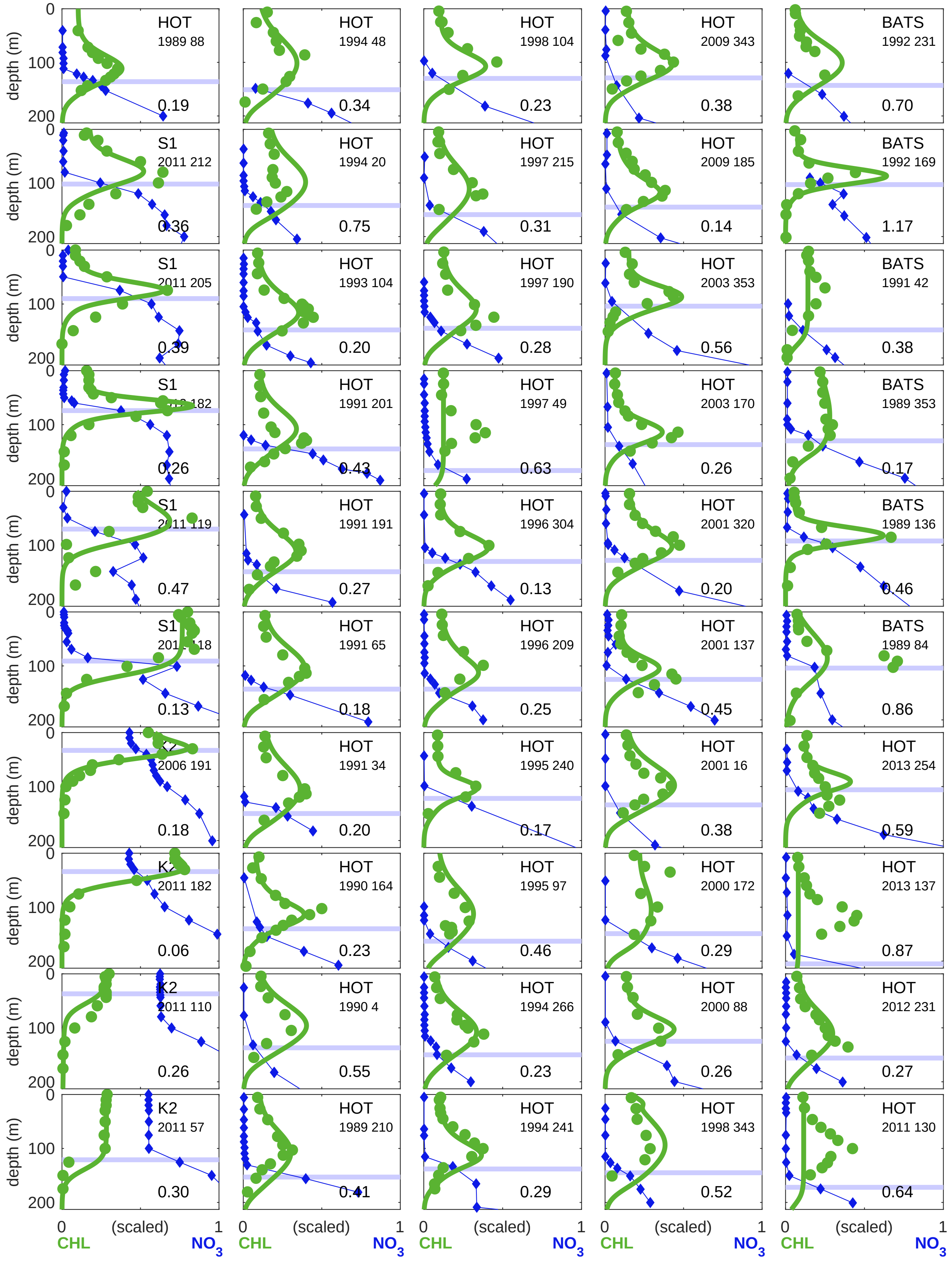
HOT 1989 88
0.19
HOT 1994 48
0.34
HOT 1998 104
0.23
HOT 2009 343
0.38
BATS 1992 231
0.70
S1 2011 212
0.36
HOT 1994 20
0.75
HOT 1997 215
0.31
HOT 2009 185
0.14
BATS 1992 169
1.17
S1 2011 205
0.39
HOT 1993 104
0.20
HOT 1997 190
0.28
HOT 2003 353
0.56
BATS 1991 42
0.38
S1
0.26
HOT 1991 201
0.43
HOT 1997 49
0.63
HOT 2003 170
0.26
BATS 1989 353
0.17
S1
0.47
HOT 1991 191
0.27
HOT 1996 304
0.13
HOT 2001 320
0.20
BATS 1989 136
0.46
S1
0.13
HOT 1991 65
0.18
HOT 1996 209
0.25
HOT 2001 137
0.45
BATS 1989 84
0.86
2006 191
0.18
HOT 1991 34
0.20
HOT 1995 240
0.17
HOT 2001 16
0.38
HOT 2013 254
0.59
2011 182
0.06
HOT 1990 164
0.23
HOT 1995 97
0.46
HOT 2000 172
0.29
HOT 2013 137
0.87
K2 2011 110
0.26
HOT 1990 4
0.55
HOT 1994 266
0.23
HOT 2000 88
0.26
HOT 2012 231
0.27
K2 2011 57
0.30
HOT 1989 210
0.41
HOT 1994 241
0.29
HOT 1998 343
0.52
HOT 2011 130
0.64
depth (m)
0
100
200
0
(scaled)
1
CHL
NO3

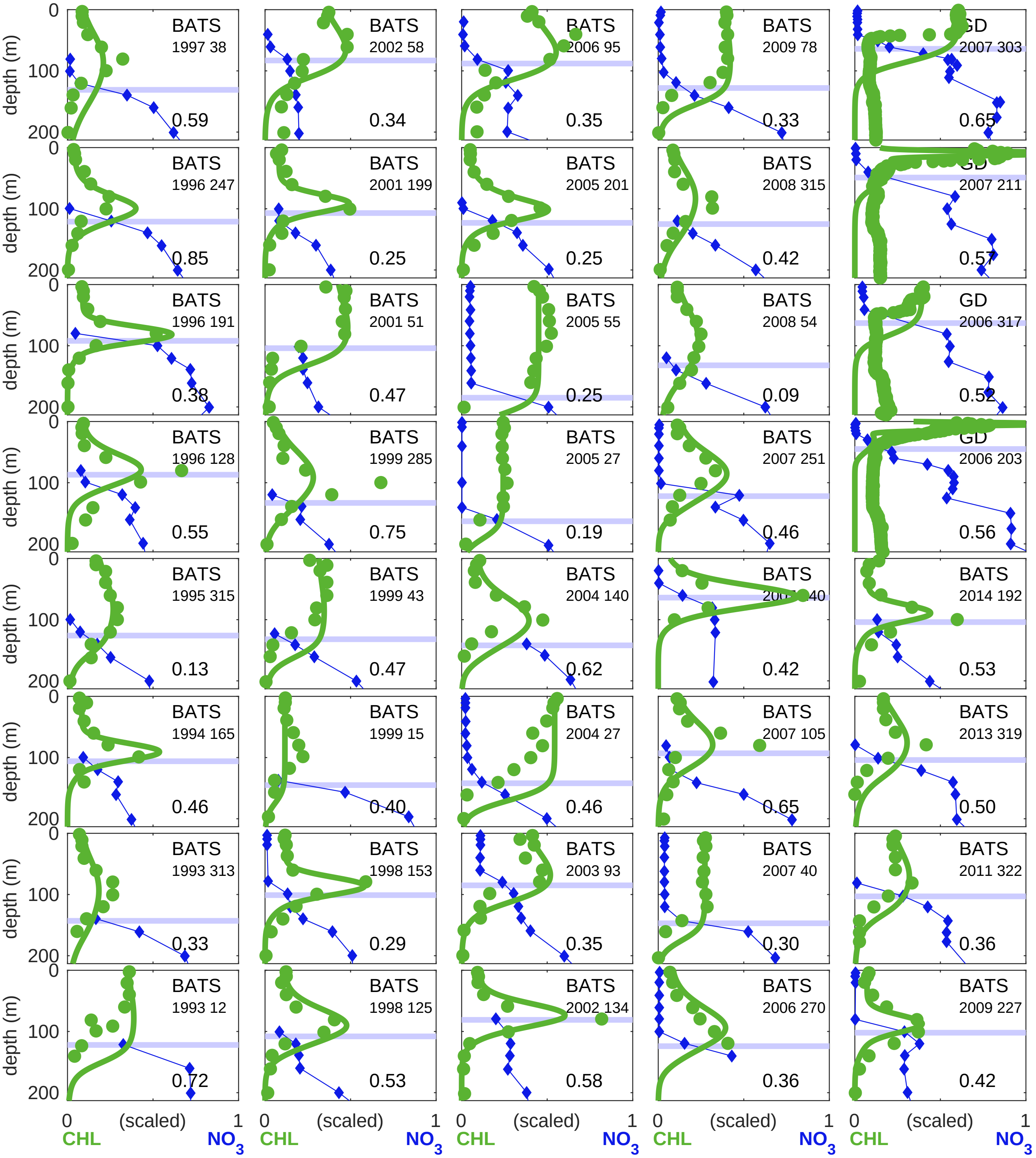

Figure S2. Selected profiles of CHL (green) and NO_3 (blue), as observed (symbols) and CHL hindcasted by our algorithm (green line). Values are normalized, for CHL by 0.7mg-CHLm^{-3} (K2:1.6, GD:2.2), for NO_3 by 5mmol-Nm^{-3} (K2:40). Observations originate from the Bermuda Atlantic Time-Series (BATS), Hawaii Ocean Time-series (HOT), two North Pacific stations, subarctic K2 and subtropical S1, and Gotland Deep (GD). Horizontal lines indicate the chemocline depth z_N. Year, Julian day, and relative RMS deviations of the model are added. The distribution of RMS errors was binned for each station and the number of profiles sampled randomly from each segment was set proportional to the total number in that segment so that the random selection of profiles/dates preserves the error statistics.

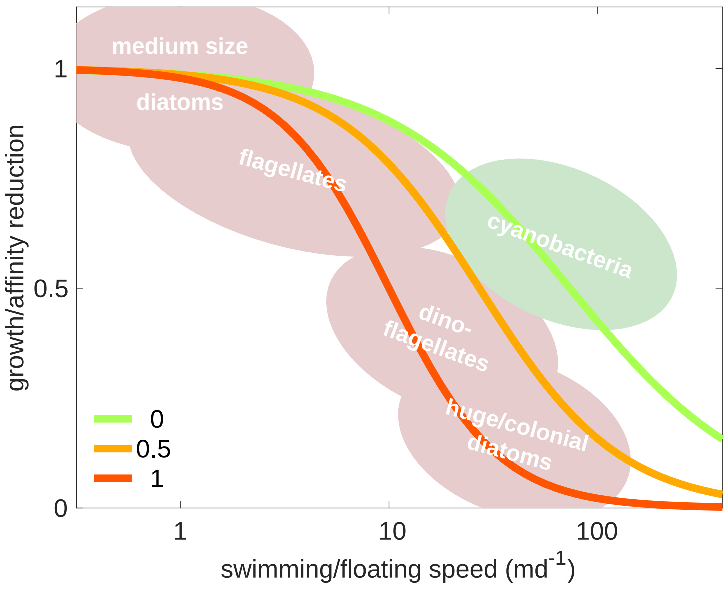

Figure S3. Trade-off in physiological performance with increasing motility (Eq. (3)), shown for three values of ε denoting the strength of the physiological trade-off. The colored areas display estimated ranges from literature[7–9].

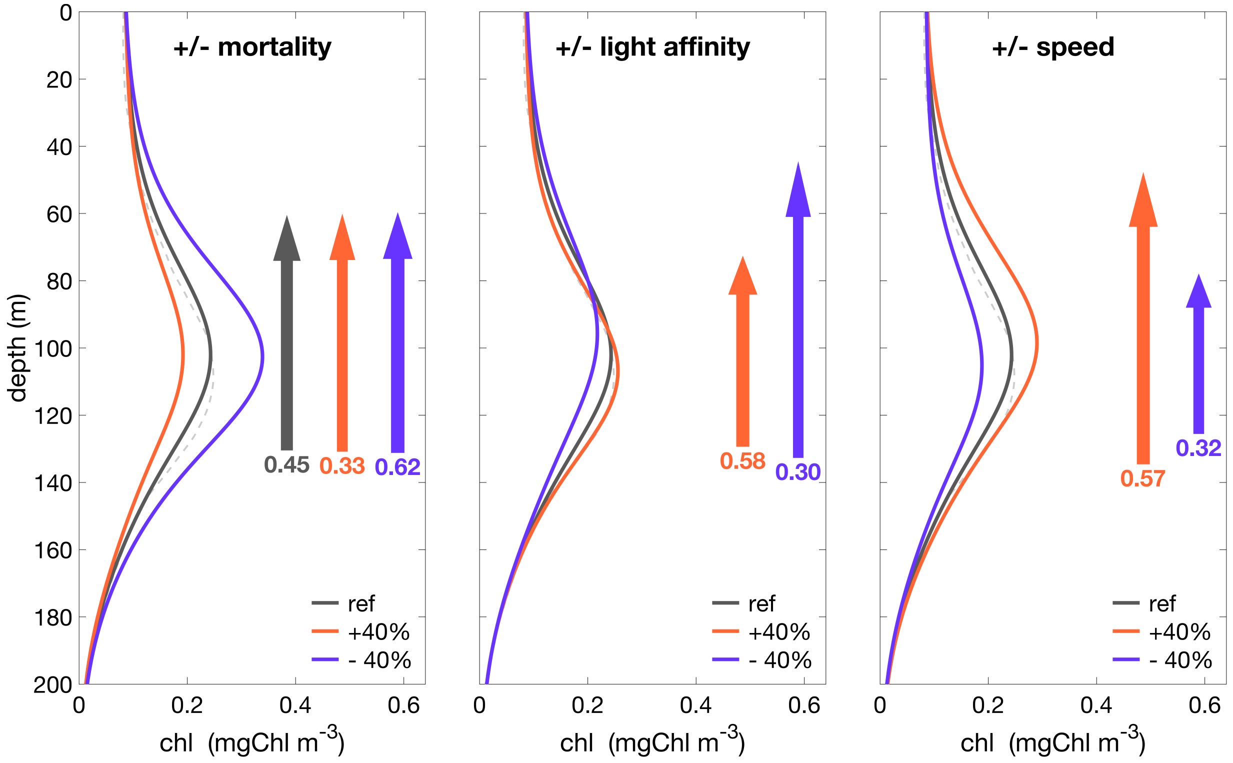

Figure S4. Sensitivity of the average CHL profile and biological N-flux simulated for station HOT in the period 1989-2014 to changes in model parameters. From left: Specific density dependent mortality m (Eq. (6)), specific light affinity α (Eq. (1)), and critical speed v^* in the physiological trade-off Eq. (4) are varied by 40% up and down. For comparison, the reference run (dark grey) and the mean of observations (dashed light grey) profiles are shown. Arrows and numbers display the average nutrient release (mmol-N m^{-2}d^{-1}) by active movers working as an upward pump (cf. Fig. 3).

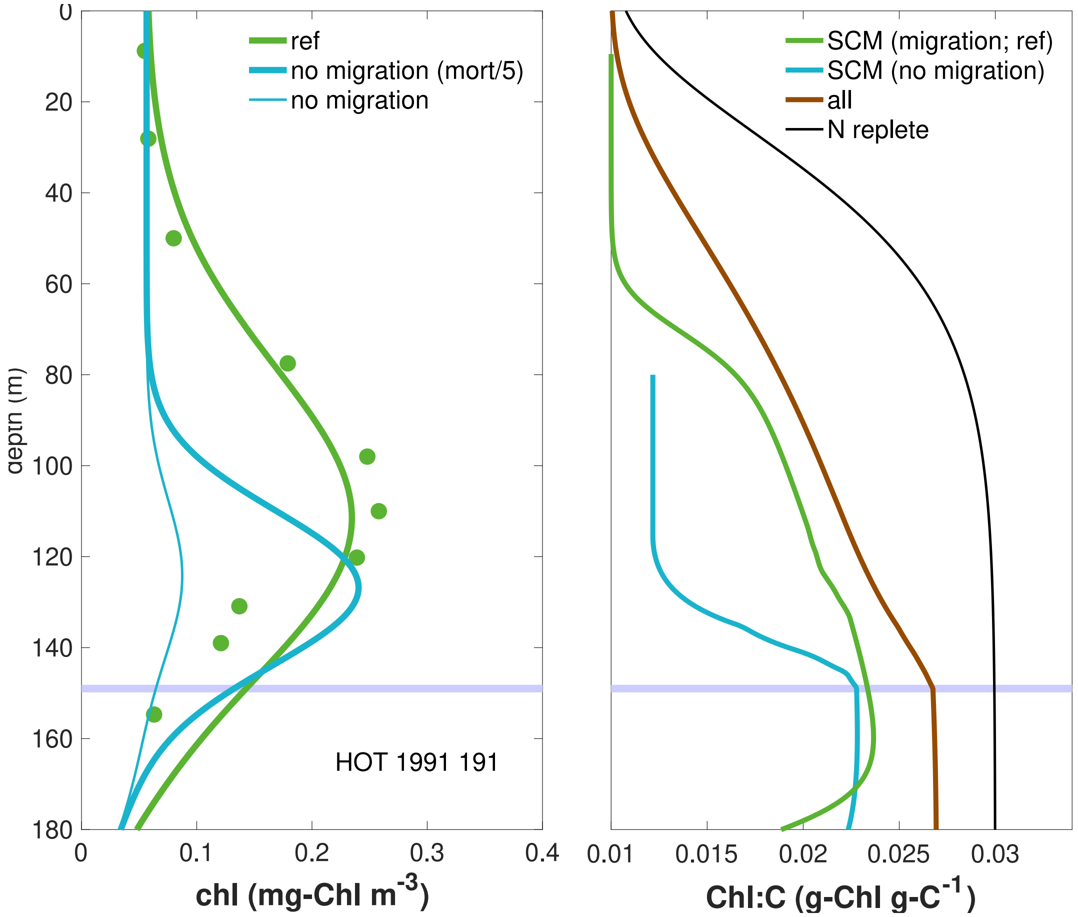

Figure S5. Left: Chl profiles for summer 1991 at HOT, as observed (circles) or simulated with (”ref”, green line) or without migration (thin bluegreen line). To compensate for the reduced growth capability of non-migratory phytoplankton, the specific mortality rate has been lowered by a factor of 5 (thick bluegreen line). The position of the chemocline is indicated with a purple line. Right: Chl:C ratios calculated for different populations, the migratory phytoplankton (green), a non-migratory building the SCM (bluegreen), the entire population (surface immobile + migratory SCM, brown), and for the entire population assuming N-replete cells, thus expressing exclusively photo–acclimation (black).

References

1. Siegel, D. *et al.* Bio-optical modeling of primary production on regional scales: the Bermuda BioOptis project. *Deep Sea Res.* **48**, 1865–1896 (2001).
2. Schanz, F., Senn, P. & Dubinsky, Z. Light absorption by phytoplankton and the vertical light attenuation: ecological and physiological significance. *Oceanography and Marine Biology* **35**, 71–96 (1997).
3. Chen, B. & Smith, S. L. Optimality-based approach for computationally efficient modeling of phytoplankton growth, chlorophyll-to-carbon, and nitrogen-to-carbon ratios. *Ecol. Mod.* **385**, 197–212 (2018).
4. Bissinger, J. E., Montagnes, D. J., Harples, J. & Atkinson, D. Predicting marine phytoplankton maximum growth rates from temperature: Improving on the Eppley curve using quantile regression. *Lim. Ocean.* **53**, 487–493 (2008).
5. Zubkov, M. V. Faster growth of the major prokaryotic versus eukaryotic CO_2 fixers in the oligotrophic ocean. *Nat.comm.* **5**, 3776 (2014).
6. Richardson, K. & Bendtsen, J. Vertical distribution of phytoplankton and primary production in relation to nutricline depth in the open ocean. *Mar. Ecol. Progr. Ser.* **620**, 33–46 (2019).
7. De Nobel, W., Matthijs, H. C., von Elert, E. & Mur, L. R. Comparison of the light-limited growth of the nitrogen-fixing cyanobacteria *Anabaena* and *Aphanizomenon*. *New Phytol.* **138**, 579–587 (1998).
8. Finkel, Z. V. *et al.* Phytoplankton in a changing world: cell size and elemental stoichiometry. *J. Plankton Res.* **32**, 119–137 (2010).
9. Wirtz, K. W. Non-uniform scaling in phytoplankton growth rate due to intracellular light and CO_2 decline. *J. Plankton Res.* **33**, 1325–1341 (2011).
